# Traceability and comparability through crosswalks with the NeuroMET Memory Metric

**DOI:** 10.1038/s41598-023-32208-0

**Published:** 2023-03-30

**Authors:** J. Melin, S. J. Cano, A. Gillman, S. Marquis, A. Flöel, L. Göschel, L. R. Pendrill

**Affiliations:** 1 Division Safety and Transport, Division Measurement Science and Technology, RISE, Research Institutes of Sweden, Gothenburg, Sweden; 2 Modus Outcomes Ltd, 4th Floor St. James House, St. James Square, Cheltenham, GL50 3PR England, UK; 3 Modus Outcomes LLC, CIC, 1 Broadway, 14th Floor, Cambridge, MA 02142 USA; 4 Department of Neurology, University Medicine Greifswald, Greifswald, Germany; 5 German Center for Neurodegenerative Diseases (DZNE), Standort Rostock/Greifswald, Germany; 6 Charité – Universitätsmedizin Berlin, corporate member of Freie Universität Berlin and Humboldt Universität zu Berlin, Department of Neurology, Charitéplatz 1, 10117 Berlin, Germany; 7 Charité – Universitätsmedizin Berlin, corporate member of Freie Universität Berlin and Humboldt Universität zu Berlin, NeuroCure Clinical Research Center, Charitéplatz 1, 10117 Berlin, Germany

**Keywords:** Cognitive neuroscience, Outcomes research

## Abstract

Accurate assessment of memory ability for persons on the continuum of Alzheimer’s disease (AD) is vital for early diagnosis, monitoring of disease progression and evaluation of new therapies. However, currently available neuropsychological tests suffer from a lack of standardization and metrological quality assurance. Improved metrics of memory can be created by carefully combining selected items from legacy short-term memory tests, whilst at the same time retaining validity, and reducing patient burden. In psychometrics, this is known as “crosswalks” to link items empirically. The aim of this paper is to link items from different types of memory tests. Memory test data were collected from the European EMPIR NeuroMET and the SmartAge studies recruited at Charité Hospital (Healthy controls n = 92; Subjective cognitive decline n = 160; Mild cognitive impairment n = 50; and AD n = 58; age range 55–87). A bank of items (n = 57) was developed based on legacy short-term memory items (i.e., Corsi Block Test, Digit Span Test, Rey’s Auditory Verbal Learning Test, Word Learning Lists from the CERAD test battery and Mini Mental State Examination; MMSE). The NeuroMET Memory Metric (NMM) is a composite metric that comprises 57 dichotomous items (right/wrong). We previously reported on a preliminary item bank to assess memory based on immediate recall, and have now demonstrated direct comparability of measurements generated from the different legacy tests. We created crosswalks between the NMM and the legacy tests and between the NMM and the full MMSE using Rasch analysis (RUMM2030) and produced two conversion tables. Measurement uncertainties for estimates of person memory ability with the NMM across the full span were smaller than all individual legacy tests, which demonstrates the added value of the NMM. Comparisons with one (MMSE) of the legacy tests showed however higher measurement uncertainties of the NMM for people with a very low memory ability (raw score ≤ 19). The conversion tables developed through crosswalks in this paper provide clinicians and researchers with a practical tool to: (i) compensate for ordinality in raw scores, (ii) ensure traceability to make reliable and valid comparisons when measuring person ability, and (iii) enable comparability between test results from different legacy tests.

Alzheimer’s Disease (AD) is best conceptualized as an inherently complex continuum of cognitive impairments^1^. The transition from the preclinical stage of AD, including the length of time and whether or not an individual will become symptomatic, remains unclear^1^. In addition, detecting and potentially treating individuals who may eventually develop AD is both operationally and conceptually challenging^2^. While biomarkers such as Aβ (measured by positron emission tomography (PET) imaging or CSF assays) have been transformative for the detection of pre-clinical AD, their invasiveness, high cost and lack of availability further ^3^ increases the need for a global and cost-effective solution.

Cognitive decline is the cardinal sign of disease progression for AD patients. Neuropsychological tests are currently used to measure cognition and have been used to: estimate risk of disease development; predict disease progression; and monitor therapeutic interventions^1,4^. However, individuals who report subjective cognitive decline (SCD) may still perform within the normal range on currently available neuropsychological tests^5^, despite a growing body of research which shows that these individuals may represent the first symptomatic stage of the AD continuum^6,7^. In addition, measurement of cognition is currently limited by a lack of standardization and metrological quality assurance, as well as a multitude of measures that lack a common frame of reference^8^. This compromises the comparison of data sets and our ability to detect meaningful changes in individual patients along the AD continuum. Therefore, there is a growing need for better, reliable measurement, especially at the early stages of cognitive decline.

Traditional psychometric methods (i.e., based on classical test theory) do not account for the ordinal nature of data generated by human responses, and lack the ability to separate person ability and item difficulty^9–12^. Not accounting for these two aspects when analysing cognitive data has a direct impact on clinicians’ and researchers’ abilities to make inferences about current statues, diagnoses, management, and treatment throughout the healthcare system. In contrast, the Rasch model is a ‘*specifically metrological approach to human-based measurement’* (p.28, 13), which can compensate for the ordinality of data and provide separate estimates of person and item attributes. In turn, this allows for standardization and measurement quality assurance for cognitive measures in the same manner as already adopted and implemented approaches for regular SI quantities and units, thus providing clinicians and researchers with better possibilities to make reliable and valid decisions in healthcare.

Metrological traceability is defined as ‘*the property of a measurement result related to a reference through a documented unbroken chain of calibrations*’^14^. Traceability is necessary for any kind of reliable and valid comparison, such as when comparing the individual’s cognitive ability against a reference value, how their cognitive ability changes (or does not change) over time, or how their cognitive ability is affected by treatment. The Rasch model is a particularly important metrological logistic regression since it enables the separation of person and item attributes^15^ from response scores, where the items can be considered as metrological references^16^. In the same way that meters can be converted to inches via crosswalks (e.g., a conversion table), cognition measured with different tests can be placed in the same frame of reference and metrologically compared.

Episodic memory, the ability to recall information about events of our lives^17^, is one of the first areas of cognition that is impacted in individuals with AD, and is also highly predictive of AD pathology^18^. Studies in healthy individuals who eventually progressed to an AD diagnosis have shown that decline in episodic memory is a core component of preclinical AD^19^. Previously, we developed the NeuroMET Memory Metric (NMM) to estimate episodic memory loss, following a metrological approach based on the Rasch model^20^. The NMM was generated from a bank of items carefully selected from legacy short-term memory tests, linking language- and cultural-free items (blocks, digits) to more complex word recalling items^21^. Our technical report on the development of the NMM shows it is well suited for a cohort clinically spanning the AD continuum. In addition, the NMM reduces measurement uncertainties for memory ability compared with individual legacy test without jeopardizing validity^21^.

Creating a formal empirical link (known as a crosswalk) from existing legacy neuropsychological test data to the NMM, is an important and practical contribution to traceability. Thus, different memory test data can be linked to a common metric of the measurand by means of co-calibration of item parameters. This approach helps connect existing research findings based on existing memory tests to one another and in relation to the new NMM. The aim of this paper is to provide crosswalks between the legacy short-term memory tests Corsi Block Test (CBT), Digit Span Test (DST), Rey’s Auditory Verbal Learning Test (RAVLT), Word Learning List from the CERAD test battery (WLL CERAD) and Mini Mental State Examination (MMSE) from which items have been chosen to make up the NMM. The resulting crosswalk conversion tables can overcome several of the shortcomings in current practice by providing clinicians and researchers with a practical tool to: (i) compensate for ordinality in raw scores; (ii) ensure traceability to make reliable and valid comparisons when measuring person ability; and (iii) enable comparability between test results from different legacy tests.

## Methods

### Subjects and data collection

The NeuroMET cohort included individuals with subjective cognitive decline (SCD, n = 38), mild cognitive impairment (MCI, n = 28), dementia due to suspected AD (n = 27), and healthy controls (HC, n = 35) recruited from Charité Hospital between 2016 and 2022. Inclusion criteria were 55–90 years of age, normal vision with or without aid and ability to consent, further details of the cohort are described elsewhere^20^. In addition, SCD (n = 88) participants from the SmartAge study^22^ were also added to the sample for analysis.

Participants of the SCD group have expressed self-experienced persistent decline in cognitive functioning during at least 6 months and associated worries, while achieving normal cognitive results considering their age^23^. The clinical dementia rating (CDR) global score was 0.5 for MCI and ≥ 1 for AD^24^. MCI and AD patients showed objective memory impairment of around -1.5 SD (for MCI) or -2.5 SD (for AD) below age- and education-adjusted norm values in relevant cognitive tests.

Each assessment was carried out over two days. Most of the legacy tests included in the NMM were performed on the first day of each assessment (CBT, DST, WLL CERAD and MMSE), while one test was completed on the second day (RAVLT). The same study assistant was responsible for conducting most of the neuropsychological assessments.

The NeuroMET project was approved by the Ethics Committee of the Charité – Universitätsmedizin Berlin, Germany, and was conducted in accordance with the declaration of Helsinki.

### The NeuroMET Memory Metric

The NMM comprises 57 carefully selected memory items from legacy tests^21^. The process of combining the 57 memory items is reported in detail elsewhere^21^. In short, the focus was on improving targeting, maximizing reliability and minimizing measurement uncertainties by: i) selecting sets of items covering the full range of abilities, and ii) by selecting items giving most information for people with higher abilities. The set of items also had to have associated construct specification Eqs.^11,25^, which provide an comprehensive empirical understanding of how the collection of items works together; what is being measured; and how validity is ensured. Short-term memory items from the CBT forward sequence (n = 14), DST forward sequence (n = 12), the RAVLT first trial A-list (n = 15), and the WLL CERAD first trial (n = 10) were included. Additionally, the memory items (immediate recall n = 3 and delayed recall n = 3) from MMSE were included in order to enable conversion from the NMM to the MMSE (this did not affect the targeting or reliability noticeably but was done for purely practical reasons as MMSE is the most widely used cognitive test).

### Data analysis

Person responses to any of the memory items in the NMM are given a classification number, either 1 for pass or 0 for fail. These classification numbers do not have a numerical meaning, but instead serve to indicate ordered categories. Responses are related both to an individual’s ability and the difficulty of the items, thus, through measurand restitution, separate values for task difficulty and person ability can be obtained.

The dichotomous Rasch model using RUMM2030^26^ was applied to the individual legacy tests and the NMM. The extent to which the observed data fit the predictions of the Rasch model help evaluate how well the established metric adheres to fundamental principles of metrology. The Rasch model was chosen as it is a particular metrological logistic regression suitable to human-based measurements^13^ to properly transform ordinal data into stable linear measures separately for memory task difficulty and person memory ability. The Rasch model is the only item response theory model to have an additive latent model, and therefore parameter separation (compared with two parameters logistic (2PL) or three parameters logistic (3PL) item response theory models). In turn, conditional inference results in sample-free item estimates, providing invariant measurement and specific objectivity to ensure traceability.

In the development of the NMM, our analyses have been focused on targeting, conventional tests of model validity in terms of goodness of fit, differential item functioning, local dependency, dimensionality, and reliability:

- *Targeting*: By inspecting the spread of person locations (i.e., range of memory abilities in the cohort) and item locations (i.e., range of the items), targeting was assessed. There is no specific criterion^27^, but the better coverage, the better targeting and the closer the mean person location is to the mean item location indicates whether the person sample is off centered from the items.
- *Item fit*: By examining the extent to which observed data accord with the expected values that are defined by the measurement model, item fit was assessed with fit residuals and chi-square statistics. These measure the extent to which items were endorsed consistently based on their location on the continuum. Fit residual recommended bounds are between -2.5 and 2.5, and chi-square statistics were evaluated through their p-values, adjusted with Bonferroni correction for multiple testing^28^.
- *Differential item functioning (DIF)*: The invariance and the extent to which items are stable across different subgroups – here diagnosis and gender – were assessed by examining the estimated person ability differences between class intervals within the subgroups using analysis of variance (ANOVA)^29^. A significant p-value for differences between subgroups indicates DIF.
- *Local dependency (LD)*: To assess the extent of LD among items, residual correlations were evaluated against a relative cut off. They were classified as LD if the item residual correlations were greater than 0.20 above the average correlations^30^.
- *Dimensionality:* Smith’s method was applied^31^, where the positive and negative patterns in a principal component analysis (PCA) of item fit residuals define two subsets of items. This is followed by estimates of person memory abilities for each subset which are then compared using an independent t test. To support unidimensionality, the percentage of tests outside the range − 1.96 to 1.96 should not exceed 5%.
- Reliability: The person separation index (PSI) describes the proportion of true variance in the total variance of person measures^32^ and was used to assess the reliability and inform on the capacity of the items to differentiate between subgroups in the population.

**Figure 1:**
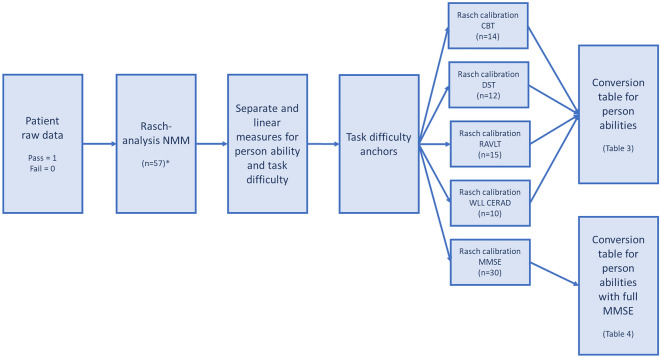
Flow-chart of raw data processing via the Rasch analysis and calibration into conversions tables and crosswalks for score-to-measures. * The full MMSE is not included in the NMM, thus, 57 is a sum of all items from other legacy tests (CBT n = 14, DST = 12, RAVLT n = 15 and WLL CERAD n = 10) and the six memory items from MMSE.

Figure 1 shows the process from raw data, via the Rasch analysis, to crosswalk tables with score-to measure conversions. In line with the Salzberger et al.^8^ methodology, crosswalks were enabled via item anchors from the NMM into separate analyses of the legacy memory tests. Score-to-measure conversions were retrieved for the NMM of 57 items and the individual legacy memory tests. Using the anchored task difficulty measures from the NMM, each location on the logit scale were matched with the closest location on the NMM measure. Six MMSE items included in the NMM were anchored, and the remaining MMSE items were scaled around them to enable a crosswalk between the NMM and the full MMSE.

Since Rasch analysis is a form of logistic regression, person abilities were derived on the logit (log-odds) scale, which has an infinite theoretical range, and an observed range of -4.74 to 3.63. To provide a more accessible interpretation^33^, the person abilities were transformed into an intuitive metric of 0 to 100 using a linear transformation, thereby preserving the interval nature of the Rasch-derived values^34^. As item locations were constrained to sum to 0, the transformation mapped 0 on the logit scale to 50 on the 0–100 scale, thereby preserving the ‘middle point’ of the scale. This method was chosen because of the relatability of a 0–100 scale in preference to other linear transforms of logit locations (such as methods using the least measurable difference (LMD), the standard error of measurement (SEM) and the least significant difference (LSD)^33,35^). Scores derived with LMD, SEM and LSD methods have a starting value of 0 but varying largest values. While it is convenient to have a starting value of 0, the interpretability of a number like 12 out of 17, for example, is much lower than a 12 out of 100. Any score on a 0–100 scale is comparable to a percentage and therefore highly accessible and user-friendly. It has been argued^33^ that a 0–100 scale may provide an inflated sense of precision, however, we counter this by providing measurement uncertainties.

### Ethics approval and consent to participate

The study was approved by the ethics committee of the Charité University Hospital, Berlin, Germany (EA1/197/16 and EA2/121/19). All participants gave written, informed consent.

## Results

### Subjects

For the final dataset for this study, a total of 360 visits were completed, comprising of assessments from HC (n = 87), individuals with SCD (n = 167), individuals with MCI (n = 52), and individuals with AD (n = 54). The assessments were almost equally distributed between men (n = 182) and women (n = 178), and the age range was 55–87 years. Table 1 provides details about characteristics and memory abilities for the total sample of individual assessments and separated by the diagnostic groups. There were no missing data for CBT and only two assessments (individuals with AD) with missing data for DST. For RAVLT, 41 assessments were missing because other versions of the tests were conducted. The SCD participants from the SmartAge study^22^ did not undergo WLL CERAD and MMSE, resulting in missing data for these tests in 88 assessments.

**Table 1:** Person characteristics and mean (SD) person ability measures (in logits) for the total sample of individual assessments and separated by the diagnostic groups.

	Total	HC	SCD	MCI	AD
	n = 360	n = 87	n = 167	n = 52	n = 54
Age, mean (SD)	70 (7)	71 (8)	68 (6)	71 (6)	74 (6)
Women, n (%)	178 (49%)	47 (54%)	88 (53%)	16 (31%)	27 (50%)
Education	15 (3)	15 (3)	16 (3)	14 (2)	15 (3)
APOEe4 carrier	137 (38%)	23 (26%)	50 (30%)	32 (62%)	32 (59%)
NMM, mean (SD)	0.53 (1.05)	0.84 (0.95)	0.75 (0.85)	0.23 (0.84)	-0.33 (1.31)
CBT, mean (SD)	− 0,50 (1.92)	− 0.35 (1.69)	0.07 (1.65)	− 0.97 (1.85)	− 1,90 (2.23)
DST, mean (SD)	0,16 (2.38)	0,69 (1.94)	0.59 (2.34)	− 0,46 (1.84)	− 1.41 (2.82)
RAVLT, mean (SD)	− 0.40 (1.04)	0.29 (1.01)	− 0.26 (0.75)	− 0.86 (0.53)	− 1.71 (0.93)
WLL CERAD, mean (SD)	− 0.18 (1.15)	0.24 (0.91)	0.21 (1.06)	− 0.55 (0.96)	− 1.08 (1.17)
MMSE full, mean (SD)	3.18 (1.53)	3.99 (0.74)	3.88 (1.51)	3.29 (0.89)	1.45 (1.37)

### Psychometric findings

**Figure 2:**
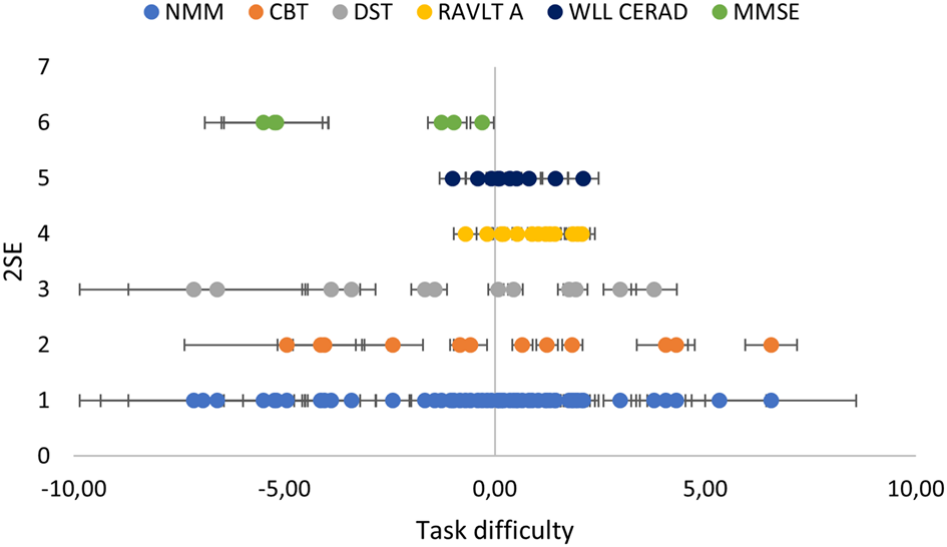
Each dot corresponds to each item’s task difficulty location (δ, x-axis) for the including items from the legacy tests. Easiest items are located to the left and the most difficult to the right. Measurement uncertainties with coverage factor of 2.

Table 2 shows a summary of overall measurement properties for the NMM and the legacy tests. A complementing illustration on the item locations (i.e., task difficulty values) can be found in Fig. 2, which outlines how CBT and DST have a wide range of items but several gaps, whereas the word lists (RAVLT and WLL CERAD) are more compact. This implies that the CBT and DST items can be used to measure person abilities for people with lower to higher abilities, but a low precision is evident due to the gaps. On the contrary, RAVLT and WLL CERAD have better precision but only if they are used for the persons located around 0.00 logits. If RAVLT or WLL CERAD are used to measure person albitites for people with lower or higher abilities, the precision decreases.

**Table 2:** Summary of measurement properties for the NMM and the legacy tests. The properties reported are based on individual analyses for the legacy tests (i.e., not when anchored to the NMM).

	NMM	CBT	DST	RAVLT A-list	WLL CERAD	MMSE 6 items	MMSE full
Number of items	57	13 *	10 *	15	10	5 *	30
Number of test persons	360	359	346	319	247	127	208
Person location range**, ***	− 4.74 (1.28) to 3.63 (1.05)	− 7.30 (2.86) to 6.84 (2.52)	− 6.29 (3.42) to 6.84 (3.62)	− 3.50 (2.63) to 3.47 (2.61)	− 3.04 (2.70) to 3.14 (2.82)	− 4.66 (2.42) to 3.99 (3.22)	− 4.62 (2.56) to 4.80 (6.62)
Item location range ***	− 6.83 (1.42) to 6.82 (2.00)	− 6.01 (0.82) to 7.14 (1.82)	− 5.12 (0.72) to 5.44 (0.70)	− 1.65 (0.28) to 1.34 (0.30)	− 1.45 (0.32) to 1.76 (0.36)	− 3.92 (1.68) to 3.18 (0.44)	− 2.34 (1.24) to 3.04 (0.36)
Number of item fit residuals ± 2.5	4	0	0	2	2	0	3
Number of items with significant chi squares	2	0	0	0	0	0	1
Number of items with significant DIF due to diagnosis	3	0	0	5	0	0	3
Number of items with significant DIF due to gender	0	0	0	0	0	0	0
Percentage of item fit residual correlations above the relative cut off	1.3%	1.3%	0%	2.9%	6.7%	10%	2.6%
Percentage of tests outside ± 1.96	19.5%	5.8%	6.7%	7.2%	2.3%	0.4%	3.4%
Person reliability (with / without extremes)	0.85 / 0.85	0.69 / 0.69	0.77 / 0.73	0.63 / 0.54	0.53 / 0.40	0.25 / 0.02	0.65 / 0.69

* CBT, DST and MMSE 6 items had one or two items that could not be estimated in the individual analyses due to extreme items.

** Person location in logits ranges include extremes. Extremes were present for DST, RAVLT A-list, WLL CERAD, MMSE 6 items and MMSE full.

*** Numbers in brackets are measurement uncertainties with coverage factor of 2.

In the NMM, four items showed item fit residuals outside the desired range of ± 2.5. Three of them originated from the “recency region” in the word lists (RAVLT item 14, 15 and WLL CERAD item 10). However, only one item showed a significant *x*^2^, and removing the items did not significantly affect the model fit. In separate papers we report further on the issue of the so-called *serial position effect* that occurs in the word learning list tests for the items in the beginning (primacy) and the end (recency) of the lists^25,36,37^.

### Crosswalks

**Figure 3:**
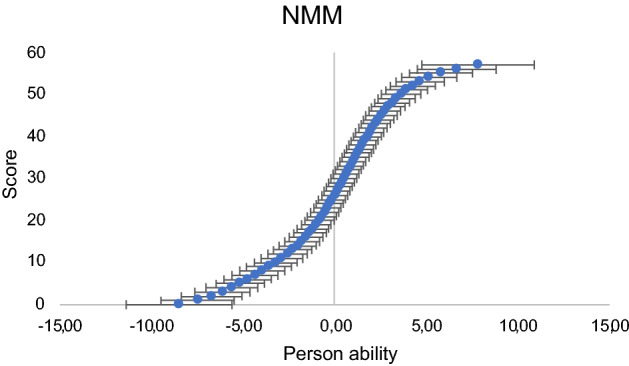
Observed S-curve correlating observed responses (raw score on the y-axis = 57) with the Rasch-estimated person ability for the NMM (x-axis). Measurement uncertainties with coverage factor k = 2.

By using the item task difficulty estimates as metrological references, conversions in the same frame of reference with estimated measurement uncertainties was enabled. Specifically, in the conversion table (Table 3) one can ‘walk’ from raw scores (i.e., counts of correct answers, ‘pass’/classification number 1) from the legacy tests, individually, and from the composite NMM to a linear measure. Figure 3 shows the correlation between the raw scores and the NMM measure for person ability, clearly indicating the ordinality in raw scores. This emphasizes the significance of using a linear measure to not underestimate persons’ abilities at the upper end of the scale or overestimate persons’ abilities at the lower end of the scale.

**Table 3:** Conversion table from NMM (57 items) to each legacy test for both raw scores and measures (in logits) and converted into a 0–100 scale. 2SE corresponds to measurement uncertainties with a coverage factor of 2.

NMM					CBT			DST			RAVLT A-list			WLL CERAD		
Score	Measure	2SE	0–100	2SE 0–100	Score	Measure	2SE	Score	Measure	2SE	Score	Measure	2SE	Score	Measure	2SE
0	− 8.49	2.86	0.00	16.83												
1	− 7.47	2.01	6.01	11.83	0	− 7.48	3.52	0	− 7.90	3.36						
2	− 6.72	1.67	10.46	9.84				1	− 6.58	2.63						
3	− 6.12	1.51	13.95	8.86	1	− 5.90	2.56									
4	− 5.61	1.40	16.96	8.27												
5	− 5.16	1.34	19.64	7.86												
6	− 4.73	1.29	22.12	7.58	2	− 4.64	2.13	2	− 4.99	2.54						
7	− 4.34	1.25	24.46	7.38												
8	− 3.96	1.23	26.71	7.22												
9	− 3.59	1.20	28.89	7.07	3	− 3.68	2.02	3	− 3.49	2.24						
10	− 3.23	1.17	31.00	6.88												
11	− 2.89	1.13	33.00	6.66												
12	− 2.57	1.09	34.87	6.40	4	− 2.68	2.05				0	− 2.59	2.73			
13	− 2.28	1.04	36.57	6.12				4	− 2.37	2.08				0	− 2.41	2.78
14	− 2.02	0.99	38.11	5.85												
15	− 1.78	0.95	39.51	5.60												
16	− 1.56	0.91	40.79	5.37	5	− 1.56	2.04				1	− 1.63	1.87			
17	− 1.36	0.88	41.97	5.18				5	− 1.35	1.98				1	− 1.43	1.93
18	− 1.18	0.85	43.06	5.00												
19	− 1.01	0.82	44.08	4.86							2	− 0.98	1.52			
20	− 0.84	0.80	45.04	4.73												
21	− 0.69	0.78	45.96	4.62										2	− 0.75	1.59
22	− 0.54	0.77	46.83	4.53	6	− 0.55	1.95				3	− 0.50	1.35			
23	− 0.39	0.76	47.68	4.45				6	− 0.42	1.92						
24	− 0.26	0.74	48.49	4.38										3	− 0.23	1.44
25	− 0.12	0.73	49.28	4.32							4	− 0.10	1.25			
26	0.01	0.73	50.06	4.27												
27	0.14	0.72	50.82	4.23												
28	0.27	0.71	51.56	4.20							5	0.25	1.19	4	0.22	1.38
29	0.39	0.71	52.30	4.18	7	0.35	1.90									
30	0.51	0.71	53.03	4.17				7	0.48	1.87	6	0.57	1.15			
31	0.64	0.71	53.75	4.16										5	0.65	1.36
32	0.76	0.71	54.48	4.16												
33	0.88	0.71	55.20	4.16							7	0.88	1.13			
34	1.01	0.71	55.93	4.17												
35	1.13	0.71	56.66	4.19							8	1.19	1.13	6	1.09	1.39
36	1.26	0.72	57.40	4.22	8	1.21	1.90									
37	1.38	0.72	58.15	4.26				8	1.32	1.85						
38	1.51	0.73	58.92	4.30							9	1.50	1.14	7	1.56	1.47
39	1.65	0.74	59.70	4.35												
40	1.78	0.75	60.51	4.41							10	1.82	1.18			
41	1.92	0.76	61.34	4.49												
42	2.07	0.78	62.19	4.58	9	2.11	1.98							8	2.11	1.63
43	2.22	0.79	63.09	4.68				9	2.16	1.87	11	2.16	1.23			
44	2.38	0.82	64.03	4.80												
45	2.55	0.84	65.03	4.94							12	2.54	1.33			
46	2.73	0.87	66.09	5.11												
47	2.93	0.90	67.23	5.31				10	3.01	1.97	13	3.00	1.50	9	2.83	1.97
48	3.14	0.94	68.47	5.55	10	3.20	2.08									
49	3.37	0.99	69.83	5.84												
50	3.63	1.05	71.36	6.18							14	3.63	1.84			
51	3.92	1.12	73.09	6.61				11	4.00	2.27				10	3.85	2.84
52	4.26	1.21	75.09	7.13	11	4.31	2.07									
53	4.66	1.33	77.44	7.80							15	4.56	2.69			
54	5.15	1.48	80.32	8.72	12	5.31	2.12	12	5.19	3.12						
55	5.78	1.72	84.01	10.10												
56	6.66	2.13	89.20	12.56	13	6.44	2.41									
57	7.83	3.05	100.00	17.98	14	7.75	3.28

**Figure 4:**
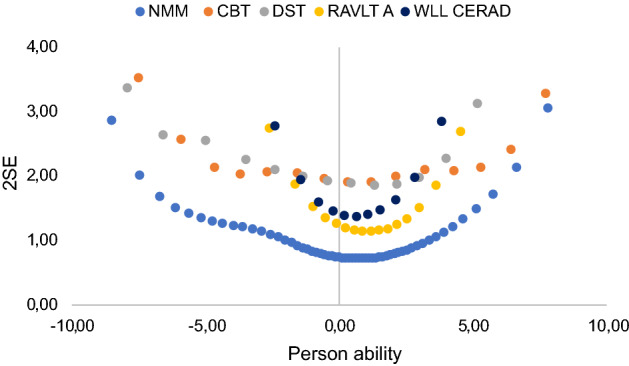
Measurement uncertainties, 2SE, on the y-axis and person ability measures (θ) on the x-axis compared for measures based on the NMM (lowest blue dots) and each of the legacy tests.

As seen in the conversion table, measurement uncertainties for person abilities are larger for all individual legacy tests compared with the NMM. This is also illustrated in Fig. 4, which also shows how measurement precision varies between the legacy tests (as described above). The NMM shows the lowest measurement uncertainties across the full span. This is a result of how the NMM has been developed to ‘fill the gaps to reach a well-targeted scale’^21^.

To provide guidance on how to read the conversion table, for instance, a person with three correct recalls on the words in RAVLT is expected to have six correct recalls in CBT. Both scores are equivalent to a measure of person ability of—0.54 ± 0.77 logits in the NMM frame of reference. Furthermore, Table 4 also provides a conversion table between the full MMSE and NMM.

**Table 4:** Conversion table from MMSE to NMM (57 items) for both raw scores and measures (in logits) and converted into the 0–100 scale for NMM in Table 3. 2SE corresponds to measurement uncertainties with a coverage factor of 2.

MMSE			NMM				
Score	Measure	2SE	Score	Measure	2SE	0–100	2SE 0–100
0	− 7.06	2.56	2	− 6.72	1.67	10.46	9.84
1	− 6.21	1.79	3	− 6.12	1.51	13.95	8.86
2	− 5.62	1.43	4	− 5.61	1.40	16.96	8.27
3	− 5.19	1.25	5	− 5.16	1.34	19.64	7.86
4	− 4.85	1.14	6	− 4.73	1.29	22.12	7.58
5	− 4.56	1.06					
6	− 4.30	1.01	7	− 4.34	1.25	24.46	7.38
7	− 4.07	0.97	8	− 3.96	1.23	26.71	7.22
8	− 3.84	0.94					
9	− 3.64	0.92	9	− 3.59	1.20	28.89	7.07
10	− 3.43	0.90					
11	− 3.24	0.89	10	− 3.23	1.17	31.00	6.88
12	− 3.05	0.88					
13	− 2.86	0.87	11	− 2.89	1.13	33.00	6.66
14	− 2.68	0.87					
15	− 2.49	0.87	12	− 2.57	1.09	34.87	6.40
16	− 2.30	0.88	13	− 2.28	1.04	36.57	6.12
17	− 2.11	0.88	14	− 2.02	0.99	38.11	5.85
18	− 1.92	0.89					
19	− 1.72	0.91	15	− 1.78	0.95	39.51	5.60
20	− 1.51	0.92	16	− 1.56	0.91	40.79	5.37
21	− 1.30	0.94	17	− 1.36	0.88	41.97	5.18
22	− 1.07	0.97	19	− 1.01	0.82	44.08	4.86
23	− 0.84	1.00	20	− 0.84	0.80	45.04	4.73
24	− 0.58	1.04	22	− 0.54	0.77	46.83	4.53
25	− 0.30	1.10	24	− 0.26	0.74	48.49	4.38
26	0.01	1.17	26	0.01	0.73	50.06	4.27
27	0.36	1.28	28	0.27	0.71	51.56	4.20
28	0.81	1.46	32	0.76	0.71	54.48	4.16
29	1.42	1.81	37	1.38	0.72	58.15	4.26
30	2.27	2.59	43	2.22	0.79	63.09	4.68

**Figure 5:**
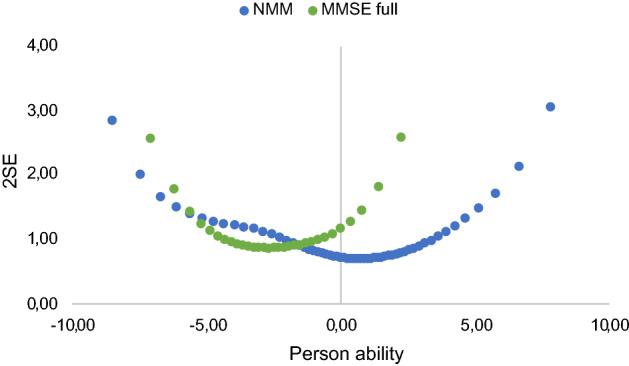
Measurement uncertainties, 2SE, on the y-axis and person ability measures (θ) on the x-axis compared for measures based on the NMM (lowest blue dots) and one (MMSE) of the legacy tests. Larger values of θ correspond to higher person ability.

Figure 5 confirms previously known issues with the MMSE when used in a healthy or early-stage population^38–40^, and the added value NMM provides. Specifically, above about -2 logits the NMM has less measurement uncertainties and better precision in measuring the person’s ability compared to the MMSE. As can be seen from the conversion table, this implies that when a patient passes on more than 20 items, regardless of which items, on MMSE, the NMM provides a more precise measure of the person’s ability.

## Discussion

In this paper we have described crosswalks between the different legacy tests included in the recently developed NMM^21^. The conversion tables presented (Tables 3–4) have been developed to provide clinicians and researchers with a practical tool to achieve three key goals:

First, as in any Rasch-transformed data set, we can compensate for ordinality in raw scores*.* Despite decades of knowledge that typical human responses ‘*have no numerical meaning and only serve to …indicate… ordered categories’* (41 p. 2), the ordinality in raw scores are still seldomly compensated for^42–45^. Tables 2 and 3 in this paper now allow researchers and clinicians to easily convert raw scores into linear measurements.

Second, we can ensure traceability to enable reliable and valid comparisons when measuring person ability. The conversion Tables 2 and 3 are built on the principles of specific objectivity and measurement invariance, which ensure metrological traceability and enable comparison of person memory ability within the same invariant frame of reference. These properties are grounded in the Rasch model, as its structure enables separate estimation of item and person parameters. Item parameters can be estimated independently (up to sample size) of the person sample using conditional inference. Item locations (within uncertainty limits) are therefore ‘invariant’, meaning that their values are not dependent on the ability of the person sample that was used for the estimation. The item locations can therefore be considered stable (again, within uncertainty limits). Consequently, with the conversion tables, which provide links from individual legacy tests to this common frame-of-reference, there is no need for re-running Rasch analyses. Rather the conversion tables will enable clinicians or researchers to interpret results that are geographically and temporally independent, and can be undertaken in different locations or times to be universally applied^46^. This is a vital development allowing clinicians and researchers to reliably measure, track over time, and compare memory ability for the future of understanding, preventing and treating patients on the AD continuum.

Third, we can enable comparability between test results from different legacy tests. The NMM was developed^21^ based on legacy test items. Legacy memory tests will continue to be used in clinical practice and research due to their relatively easy applicability, and to the long-term experience researchers have with these tests. With our crosswalk conversion tables, clinicians or researchers do not need a new testing procedure to increase the quality of their measurements. Again, by using the conversion tables (Tables 3–4), raw scores from CBT, DST, RAVLT, WLL CERAD and MMSE can be converted into the same frame of reference and in turn allow for comparisons independent of which test items are used.

Previous research has encouraged the construction of fit-for-purpose and rigorous measurements of cognitive ability^9,10^. Yet, in general, there has been little advancement in methodologies around human-based measurements over the past 60 years^47^. Specifically, there has been a call for a reappraisal of metrology to provide quality assurance and comparability via metrological traceability^48^. The development of the NMM has been a direct response to that^22^, but at the same time ‘*the approach does not propose a new method of assessing cognition, but rather a more psychometrically sound interpretation’* (49, p. 59). In addition to traceability, measurement uncertainty is a key metrological aspect. However, assessing or reporting measurement uncertainty has little tradition in human-based measurements. Within the Rasch model, the standard error of measurement (i.e., the reciprocal proportion to the square root of the amount of information) is an estimate of the random error and is typically used to reflect measurement uncertainties, while considerations of what extent systematic errors contribute to measurement uncertainty have not, as yet, been made^8,50,51^. Thus, the Rasch model provides opportunities to better align measurement uncertainty in psychometric studies with that used in physical metrology^8^. Thus, in addition to the NMM being a practical day-to-day tool, this methodological work is a direct response to better standardization and measurement quality assurance for cognitive measurements in this new area of metrology.

Furthermore, previous studies of both the ADAS-cog^52^ and the MMSE^53^ have shown that cognitive ability of those with an early cognitive decline are underestimated due to poor targeting. This may lead to problems in detecting clinical change, particularly for the preclinical phase of the AD continuum. Elsewhere, we have shown how the selected items from legacy short-term memory tests shorten overall testing time while maintaining coherence in item design, without jeopardizing validity for the NMM^21^. Specifically, as is also illustrated here in Fig. 4, the NMM shows the lowest measurement uncertainties across the full span of memory abilities compared to the legacy tests. This will allow for using one scale across the AD continuum to better understand cognitive decline and disease progression.

Nowadays, item-banks are proposed to reduce patient burden by shortening assessments by utilising computerised adaptive testing (CAT) (c.f. 54–56). CAT is a method of delivering test items that is tailored to an individual, whereby the order and difficulty of items that appear are directly related to the individuals’ previous responses. The algorithm takes into account both person ability and item difficulty, with each individual only answering the number of questions necessary to make a precise estimate of their ability with a pre-specified level of precision^57^. The next step in the NeuroMET project is to make the NMM even more user friendly, by developing an app where clinicians and researchers will be able to select either an item set or the full NMM, so that patient responses are transformed via a scoring algorithm into the memory ability measurement value. Using the NMM item-bank and the NMM app, the same individual can be measured over a period of years with a tool that is adaptive and sensitive to their cognitive decline over time. Likewise, practice effects that dilute accuracy in measures of person abilities can be diminished due to tailoring fewer items to the person’s ability.

### Limitations

There are some methodological considerations to bear in mind when interpreting the findings of this study. Firstly, as of now, the NMM has only been tested in a German population, which means that a proper assessment of metrological references has not been done and we cannot claim item-stability cross-countries for the NMM at this stage. Legacy tests with blocks and number sequences are, however, free from cultural and language bias and are traditionally used without any psychometric verification in different countries. More recently, we have made validation tests on the DST^11,58^ and RAVLT^36^ in a Swedish population which have confirmed item stability included in those tests, and further cross-country validation studies are being planned.

Secondly, the NMM items were assessed as part of an extensive battery of neuropsychological tests. Thus, the items were not assessed consecutively (all but RAVLT were conducted on the first day) and there are only selected items from the full legacy tests included, hopefully reducing the effects of exhaustion. This should be investigated in forthcoming work, but again, within the frame of the Rasch model, the item locations were estimated using a method independent of the distribution of the sample and should therefore be roughly the same within quoted uncertainties when estimated from another sample.

Thirdly, the NMM shows overall good measurement properties to measure person memory ability. For the total NMM only three items showed significant DIF with regards to sub-groups but for the individual test RAVLT as many as five of fifteen items showed DIF with regards to sub-groups. This issue has, however, been studied in detail in one of our previous papers^25^. We concluded that this may be due to dimensionality issues but refrained from separating groups of items into subtests (for RAVLT for primacy and recency items) in an attempt to improve the unidimensionality as the measurement uncertainties become too large with only a handful of dichotomous items. The overall smallness of DIF meant that we have not yet looked in detail at any Differential Test Functioning effects. Furthermore, we decided to keep all RAVLT items – despite two of them showing DIF (one from WLL CERAD) – due to negligible effects on the overall estimate of person memory ability and to offer the possibility for clinicians not used to Rasch to still relate to the composite NMM.

Finally, at present there are few other studies providing these kinds of crosswalks^8,59,60^. This implies limited recommendations, guidelines, or best practices to follow. However, this work was guided by well-known metrological underpinnings and well-established techniques for item-person separation, item anchoring and conversions. Therefore, we believe that the conversion tables for NMM and the legacy tests are valid.

## Conclusions

As a response to the call for accurate and sensitive assessment of memory abilities for persons across the AD continuum, the NMM has recently been developed based on a Rasch model together with construct specification equations. The aim of doing this was to overcome the challenges of bringing several items of distinct difficulties on a common scale in order to establish a metrologically validated memory metric.

The results of the present crosswalk study, i.e., the conversion tables for the NMM and legacy tests, now provide clinicians and researchers with a practical tool to: i) compensate for ordinality in raw scores; ii) ensure traceability to make reliable and valid comparisons when measuring person ability; and iii) enable comparability between test results from different legacy tests. By using the conversion tables presented here, better standardization and measurement quality assurance for cognitive measurements – as are already established for the regular SI quantities and units – can be enabled. We believe that the shortcomings of the use of raw scores are a major issue and the work presented here should be considered as a starting point to improve measurement quality assurance. However, further evaluations are warranted in cross-country studies, because the conversion tables are as yet only based on German data. Nevertheless, the NMM is in itself a unique metrologically validated memory metric that can be useful for early diagnosis, monitoring of disease progression and response to therapies.

## Data Availability

https://zenodo.org/record/7070958#.Yx8Cf3ZBxPY.

## Abbreviations

AD: Alzheimer’s disease
CBT: Corsi block test
CERAD: Consortium to establish a registry for Alzheimer’s disease
DST: Digit span test
EMPIR: European metrology programme for innovation and research
HC: Healthy control
IRT: Item response theory
MCI: Mild cognitive impairment
MMSE: Mini mental state examination
NMM: NeuroMET memory metric
PCA: Principal component analysis
PSI: Person separation index
RAVLT: Rey’s auditory verbal learning test
SCD: Subjective cognitive decline
WLL: Word learning list

## References

[1] Aisen PS, Cummings J, Jack CR, Morris JC, Sperling R, Frölich L (2017). On the path to 2025: understanding the Alzheimer’s disease continuum. Alzheimer’s Res. Therapy..

[2] Caselli RJ, Reiman EM (2013). Characterizing the preclinical stages of Alzheimer’s disease and the prospect of presymptomatic intervention. J. Alzheimers Dis..

[3] Alber J, Goldfarb D, Thompson LI, Arthur E, Hernandez K, Cheng D (2020). Developing retinal biomarkers for the earliest stages of Alzheimer’s disease: What we know, what we don’t, and how to move forward. Alzheimer’s & Dementia..

[4] Donohue MC, Sperling RA, Salmon DP, Rentz DM, Raman R, Thomas RG (2014). The preclinical alzheimer cognitive composite: measuring amyloid-related decline. JAMA Neurol..

[5] Jessen F (2014). Subjective and objective cognitive decline at the pre-dementia stage of Alzheimer’s disease. Eur. Arch. Psychiatry Clin. Neurosci..

[6] Amieva H, Le Goff M, Millet X, Orgogozo JM, Pérès K, Barberger-Gateau P (2008). Prodromal Alzheimer’s disease: successive emergence of the clinical symptoms. Ann. Neurol..

[7] Hong YJ, Lee JH (2017). Subjective cognitive decline and Alzheimer’s disease spectrum disorder. Dement. Neurocogn. Disord..

[8] Salzberger T, Cano S, Abetz-Webb L, Afolalu E, Chrea C, Weitkunat R, Rose J (2021). Addressing traceability of self-reported dependence measurement through the use of crosswalks. Measurement.

[9] Hobart, J. Putting the Alzheimer’s cognitive test to the test I: Traditional psychometric methods.**6**. (2013). 10.1016/j.jalz.2012.08.005 23253777

[10] Hobart J, Cano S, Posner H, Selnes O, Stern Y, Thomas R (2013). Putting the Alzheimer’s cognitive test to the test II: Rasch Measurement Theory. Alzheimer’s & Dementia..

[11] Melin J, Cano S, Pendrill L (2021). The role of entropy in construct specification equations (CSE) to improve the validity of memory tests. Entropy.

[12] Hughes LF, Perkins K, Wright BD, Westrick H (2003). Using a Rasch scale to characterize the clinical features of patients with a clinical diagnosis of uncertain, probable, or possible Alzheimer disease at intake. JAD..

[13] Pendrill L (2014). Man as a measurement instrument. NCSLI Measure..

[14] International vocabulary of metrology – Basic and general concepts and associated terms (VIM). JCGM **200**, 2012 10.1016/j.clinbiochem.2008.09.007 19863914

[15] Rasch, G. Studies in mathematical psychology: I. Probabilistic models for some intelligence and attainment tests. 184 (Nielsen & Lydiche, 1960). (Studies in mathematical psychology: I. Probabilistic models for some intelligence and attainment tests).

[16] Cano SJ, Pendrill LR, Barbic SP, Fisher WP (2018). Patient-centred outcome metrology for healthcare decision-making. J. Phys. Conf. Ser..

[17] Gallagher M, Koh MT (2011). Episodic memory on the path to Alzheimer’s disease. Curr. Opin. Neurobiol..

[18] Wagner M, Wolf S, Reischies FM, Daerr M, Wolfsgruber S, Jessen F (2012). Biomarker validation of a cued recall memory deficit in prodromal Alzheimer disease. Neurology.

[19] Langbaum JBS, Hendrix SB, Ayutyanont N, Chen K, Fleisher AS, Shah RC (2014). An empirically derived composite cognitive test score with improved power to track and evaluate treatments for preclinical Alzheimer’s disease. Alzheimers Dement..

[20] Quaglia M, Cano S, Fillmer A, Flöel A, Giangrande C, Göschel L (2021). The NeuroMET project: metrology and innovation for early diagnosis and accurate stratification of patients with neurodegenerative diseases. Alzheimer’s Dement..

[21] Melin J, Cano SJ, Flöel A, Göschel L, Pendrill LR (2022). Metrological advancements in cognitive measurement: a worked example with the NeuroMET memory metric providing more reliability and efficiency. Meas. Sens..

[22] Wirth M, Schwarz C, Benson G, Horn N, Buchert R, Lange C (2019). Effects of spermidine supplementation on cognition and biomarkers in older adults with subjective cognitive decline (SmartAge)-study protocol for a randomized controlled trial. Alzheimers Res. Ther..

[23] Jessen F, Amariglio RE, Buckley RF, van der Flier WM, Han Y, Molinuevo JL (2020). The characterisation of subjective cognitive decline. Lancet Neurol..

[24] Morris JC (1993). The clinical dementia rating (CDR): current version and scoring rules. Neurology.

[25] Melin J, Cano S, Flöel A, Göschel L, Pendrill L (2022). The role of entropy in construct specification equations (CSE) to improve the validity of memory tests: extension to word lists. Entropy.

[26] Andrich, D., Sheridan, B. S. & Lou, G. In: *Rumm 2030: Rasch Unidimensional Measurement Models*. (RUMM Laboratory, 2009).

[27] Cleanthous S, Bongardt S, Marquis P, Stach C, Cano S, Morel T (2021). Psychometric analysis from EMBODY1 and 2 clinical trials to help select suitable fatigue pro scales for future systemic lupus erythematosus studies. Rheumatol. Therapy.

[28] Hobart J, Cano S (2009). Improving the evaluation of therapeutic interventions in multiple sclerosis: the role of new psychometric methods. Health Technol. Assess..

[29] Andrich D, Hagquist C (2012). Real and artificial differential item functioning. J. Educ. Behav. Statist..

[30] Christensen KB, Makransky G, Horton M (2017). Critical values for Yen’s Q 3: identification of local dependence in the rasch model using residual correlations. Appl. Psychol. Meas..

[31] Smith RM (1996). A comparison of methods for determining dimensionality in Rasch measurement. Struct. Equ. Modeling.

[32] Andrich D (1982). An index of person separation in latent trait theory, the traditional KR.20 index, and the Guttman scale response pattern. Educ. Res. Perspect..

[33] Ekstrand J, Westergren A, Årestedt K, Hellström A, Hagell P (2022). Transformation of Rasch model logits for enhanced interpretability. BMC Med. Res. Methodol..

[34] Harwell MR, Gatti GG (2001). Rescaling ordinal data to interval data in educational research. Rev. Educ. Res..

[35] Wright, B. & Stone, M. Best test design. **1979**.

[36] Melin, J., Kettunen, P., Wallin, A. & Pendrill, L. Entropy-based explanations of serial position and learning effects in ordinal responses to word list tests. (2022).

[37] Melin, J. & Pendrill, L. A novel metrological approach to a more consistent way of defining and analyzing memory task difficulty in word learning list tests with repeated trials. In: *Proceedings of the RaPID Workshop - Resources and ProcessIng of linguistic, para-linguistic and extra-linguistic Data from people with various forms of cognitive/psychiatric/developmental impairments - within the 13th Language Resources and Evaluation Conference* 17–21 (European Language Resources Association, 2022). Available from: https://aclanthology.org/2022.rapid-1.3 (2022).

[38] Arevalo-Rodriguez I, Smailagic N, Roqué-Figuls M, Ciapponi A, Sanchez-Perez E, Giannakou A, Pedraza OL, Cosp XB, Cullum S (2021). Mini-Mental State Examination (MMSE) for the early detection of dementia in people with mild cognitive impairment (MCI). Cochrane Database Syst. Rev..

[39] Carnero-Pardo C (2014). Should the Mini-Mental State Examination be retired?. Neurología (English Edition)..

[40] Larner AJ (2018). Mini-mental state examination: diagnostic test accuracy study in primary care referrals. Neurodegener. Dis. Manag..

[41] Turetsky V, Bashkansky E (2022). Ordinal response variation of the polytomous Rasch model. Metron.

[42] Wright BD (1997). A history of social science measurement. Educ. Meas. Issues Pract..

[43] Tesio L (2003). Measuring behaviours and perceptions: Rasch analysis as a tool for rehabilitation research. J. Rehabil. Med..

[44] Wright BD, Linacre JM (1989). Observations are always ordinal; measurements, however, must be interval. Arch. Phys. Med. Rehabil..

[45] Grimby G, Tennant A, Tesio L (2012). The use of raw scores from ordinal scales: Time to end malpractice?. J. Rehabil. Med..

[46] Cano SJ, Pendrill LR, Melin J, Fisher WP (2019). Towards consensus measurement standards for patient-centered outcomes. Measurement.

[47] McKenna SP, Heaney A, Wilburn J (2019). Measurement of patient-reported outcomes. 2: Are current measures failing us?. J. Med. Econ..

[48] McGrane JA (2015). Stevens’ forgotten crossroads: the divergent measurement traditions in the physical and psychological sciences from the mid-twentieth century. Front. Psychol..

[49] Posner HB, Cano S, Carrillo MC, Selnes O, Stern Y, Thomas RG (2013). Establishing the psychometric underpinning of cognition measures for clinical trials of Alzheimer’s disease and its precursors: a new approach. Alzheimer’s Dementia.

[50] Pendrill LR, Fisher WP (2015). Counting and quantification: comparing psychometric and metrological perspectives on visual perceptions of number. Measurement.

[51] Andrich D, Pedler P (2019). A law of ordinal random error: The Rasch measurement model and random error distributions of ordinal assessments. Measurement.

[52] Cano SJ, Posner HB, Moline ML, Hurt SW, Swartz J, Hsu T (2010). The ADAS-cog in Alzheimer’s disease clinical trials: psychometric evaluation of the sum and its parts. J. Neurol. Neurosurg. Psychiatry.

[53] Pendrill LR (2018). Assuring measurement quality in person-centred healthcare. Meas. Sci. Technol..

[54] Barney M, Fisher WP (2016). Adaptive measurement and assessment. Annu. Rev. Organ. Psychol. Organ. Behav..

[55] Wainer H, Dorans NJ (2000). Computerized Adaptive Testing: A Primer.

[56] Pesudovs K (2010). Item banking: a generational change in patient-reported outcome measurement. Optom. Vis. Sci..

[57] Murray AL, Vollmer M, Deary IJ, Muniz-Terrera G, Booth T (2021). Assessing individual-level change in dementia research: a review of methodologies. Alzheimer’s Res. Therapy.

[58] Melin J, Pendrill LR, Fisher Jr William P, Cano SJ (2023). The role of construct specification equations and entropy in the measurement of memory. Person-Centered Outcome Metrology: Principles and Applications for High Stakes Decision Making.

[59] Lambert SD, Clover K, Pallant JF, Britton B, King MT, Mitchell AJ (2015). Making sense of variations in prevalence estimates of depression in cancer: a co-calibration of commonly used depression scales using rasch analysis. J. Natl. Compr. Canc. Netw..

[60] Rouse M, Twiss J, McKenna SP (2016). Co-calibrating quality-of-life scores from three pulmonary disorders: implications for comparative-effectiveness research. J. Med. Econ..

